# An Improved 3D OPC Method for the Fabrication of High-Fidelity Micro Fresnel Lenses

**DOI:** 10.3390/mi14122220

**Published:** 2023-12-09

**Authors:** Fei Peng, Chao Sun, Hui Wan, Chengqun Gui

**Affiliations:** 1 Institute of Technological Sciences, Wuhan University, Wuhan 430072, China; 2020106520010@whu.edu.cn (F.P.); 2018300003075@whu.edu.cn (C.S.); wanhui_hb@whu.edu.cn (H.W.); 2 Hubei Key Laboratory of Electronic Manufacturing and Packaging Integration, Wuhan University, Wuhan 430072, China

**Keywords:** 3D lithography, 3D OPC, Fresnel lens, PSNR

## Abstract

Based on three-dimensional optical proximity correction (3D OPC), recent advancements in 3D lithography have enabled the high-fidelity customization of 3D micro-optical elements. However, the micron-to-millimeter-scale structures represented by the Fresnel lens design bring more stringent requirements for 3D OPC, which poses significant challenges to the accuracy of models and the efficiency of algorithms. Thus, a lithographic model based on optical imaging and photochemical reaction curves is developed in this paper, and a subdomain division method with a statistics principle is proposed to improve the efficiency and accuracy of 3D OPC. Both the simulation and the experimental results show the superiority of the proposed 3D OPC method in the fabrication of Fresnel lenses. The computation memory requirements of the 3D OPC are reduced to below 1%, and the profile error of the fabricated Fresnel lens is reduced 79.98%. Applying the Fresnel lenses to an imaging system, the average peak signal to noise ratio (PSNR) of the image is increased by 18.92%, and the average contrast of the image is enhanced by 36%. We believe that the proposed 3D OPC method can be extended to the fabrication of vision-correcting ophthalmological lenses.

## 1. Introduction

Three-dimensional lithography based on laser direct writing, or mask-less lithography, refers to the process of the selective exposure of a thick photoresist from the features of the computer-aided design (CAD) model. This technique can effectively modulate the light intensity on the surface and inside the photoresist, in order to obtain a three-dimensional photoresist pattern after development. The characteristics of a smaller volume, lower manufacturing costs, and fast manufacturing efficiency make it widely used in two-dimensional (2D) micro-manufacturing applications, such as microfluidics [1], meta-materials [2], and reticles for X-ray lithography [3,4]. Recently, the flexibility of LDW-based free form grayscale mask design has attracted widespread attention and rapid development in the potential of three-dimensional (3D) microfabrication. Examples include customized micro-lens arrays (MLA) for wave-front sensing [5,6], virtual reality/augmented reality displays [7,8], super hydrophobic lenses for the humid outdoor environment [9], bionic compound eye lenses [10,11] for zoom imaging, Fresnel lenses for visual correction [12,13], Fresnel zone plates for lensless imaging [14,15], and achromatic lenses based on the Fresnel design [16,17,18,19]. All the integrated optical devices described above require collaborative optimization in design and manufacturing. Therefore, models based on different mechanisms are proposed and used to predict the microstructural morphology—for example, time- and space-based molecular diffusion models [20], 3D models based on resin cross-linking [21], kinetics-based dill exposure models [22,23,24], and 3D models based on optical proximity effects [25,26]. It is worth noting that the first two methods mentioned above, starting from more microscopic photochemical reactions and spatio-temporal effects, are more scientific and rigorous, and they have achieved significant results in multi-photon lithography. In grayscale 3D lithography, the optical proximity correction (OPC) 3D model is commonly used to compensate for the morphology of 3D structures. Although this model weakens the influence of time and space in predicting the morphology, the engineering approximation between the exposure energy and structural morphology effectively compensates for lithographic morphology errors. However, when simulating millimeter-scale structures, the existing 3D OPC method requires a huge amount of computing power and terabyte memory storage. Thus, it is necessary to develop a 3D OPC method that requires fewer computational resources, while retaining the high geometric fidelity of microstructures.

**Figure 1:**
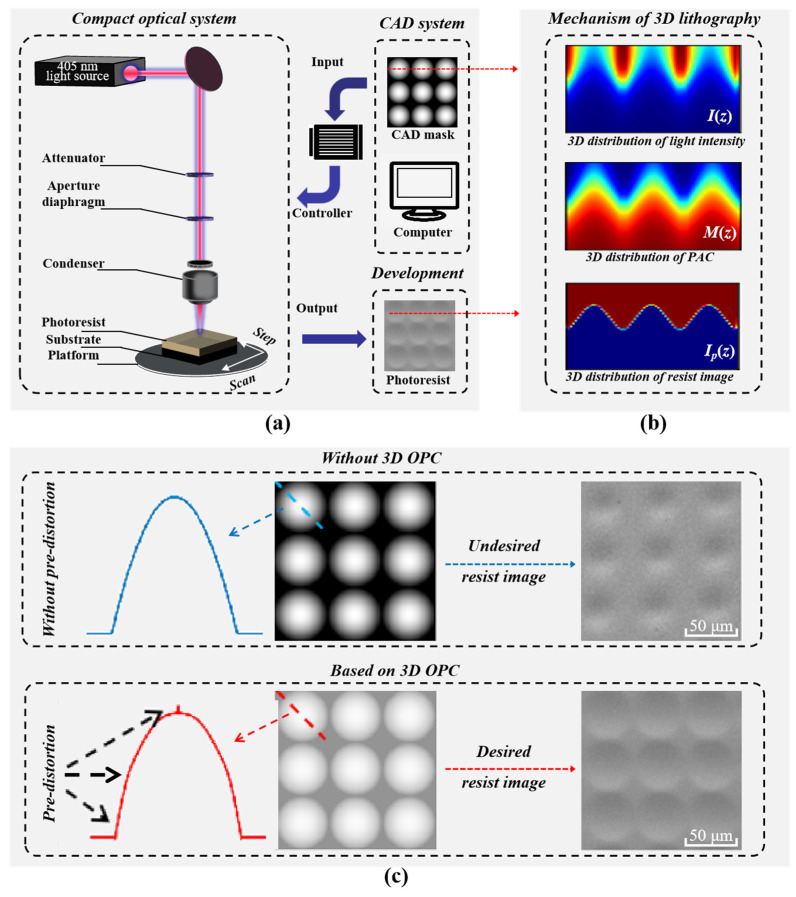
(**a**) Schematic of 3D lithography system, in which a 405 nm light source is used and the layout on the CAD mask is transferred to the photoresist by a scanning exposure and development process. (**b**) The 3D distribution of light intensity and photoactive compound concentration (PAC) in the photoresist is changed by selective exposure, and the 3D photoresist pattern is formed after development. (**c**) The CAD mask compensation method is based on three-dimensional optical proximity correction (3D OPC), where the pixelated mask is numerically solved by convex optimization to improve the print fidelity.

Figure 1a illustrates the schematic diagram of a 3D lithography system with 405 nm illumination. First, the CAD mask carrying the microstructure layout is uniformly sampled, and the exposure scheme is generated. Second, the optical signal is transferred to the photoresist-covered substrate using an electrical modulation system, and the platform scans and steps at a constant speed. Finally, the photoresist patterns are formed after a series of developing processes. Taking the red dotted line of the CAD mask as an example, the grayscale 3D lithography mechanism based on LDW is shown in Figure 1b. The selective exposure on the surface of the photoresist causes a change in the light intensity in z-direction I(z), and the photochemical reaction results in the response of the photoactive compound concentration (PAC) in z-direction M(z). As a result, a 3D photoresist image *Ip*(z) is obtained after the development process. However, the photoresist image is distorted due to the optical proximity effect (OPE) and the photochemical effect. Figure 1c shows the compensation effect of 3D OPC, which improves the fidelity of the photoresist pattern by pre-distorting the CAD mask pattern and inserting assistant features around the structure. Recently, the model-based OPC has been proposed to improve the lithographic fidelity [27,28]; the regularization term with exposed dosage [29] and interior-point optimization with a barrier function [30] have been developed to accelerate optimization; and the neural network-based compensation method [31] has been researched for the prediction of CAD masks. All these methods provide superior theories for later generations and become essential to compensate for the undesired distortions of lithography, but the computational and storage efficiency is still unacceptable. For example, in an N×N OPC problem, the derivative calculation requires $N^{2}\times N^{2}$ matrix storage. The OPC of projection lithography also has computational efficiency problems, and a series of algorithms have been developed. The development of these algorithms can serve as guidelines for the investigation of 3D OPC. Examples of such algorithms are the conjugate gradient methods [32,33], the augmented Lagrangian methods [34], the compressive sensing methods [35,36], the semi-implicit methods [37,38], and the model-driven neural network methods [39,40]. Inspired by these algorithms, Peng et al. simplified the derivatives as a matrix form and proposed the 3D OPC method based on 3D lithography [41]. Jidling et al. focused on the memory efficiency and a constrained gradient search method (L-BFGS-B) with pattern segmentation was proposed [42]. At the same time, Freymann et al. optimized the resin cross-linked 3D model based on the Downhill Simplex Algorithm (DSA) [21], which also reduced the memory requirements and computational resources. However, these algorithms still require substantial memory and computational resources, especially when optimizing at the millimeter scale. In addition, the existing 3D OPC models do not consider the interplay between optical and photochemical reaction processes, leading to inaccurate model results. Therefore, it is necessary to develop an algorithm that accurately models and optimizes such complex designs and can effectively utilize computing and memory resources.

With the development of optical devices towards flatness and customization, the Fresnel design has garnered attention from researchers, because the Fresnel design enables the creation of lightweight devices while maintaining a significantly larger field of view (FOV) [14,15,16,17]. Moreover, the advancement of back-end computational models and algorithms has greatly improved the imaging quality of Fresnel designs, leading to their widespread application [18,19]. Considering the potential of the Fresnel structure’s applications and its geometrical profile properties, this paper focuses on the establishment of numerical models for 3D OPC and the optimization of central symmetric structures based on the Fresnel design. In particular, the imaging model can be described as the convolution operation of the point spread function (PSF) and CAD mask [43,44,45]. The photochemical reaction of a thick resist can be described by the Dill model [22,23,24], which considers the change in the refractive index of the photoresist during exposure, the energy absorption in the photoresist, and the concentration distribution of photosensitivity. Thus, the nonlinear relationship between aerial images and printed photoresist images can be calibrated. To deal with the memory requirements, a subdomain division method with statistics is proposed, which combines the principle of statistics and perceives the overall region through subdomains. Subsequently, total variation (TV) is used during optimization to ensure the continuity of the subdomains. After optimization, the subdomain-based CAD mask is searched, and the optimal global CAD mask is perceived. Finally, the Fresnel lenses are transferred onto PDMS, and the transferred concave Fresnel lenses are applied in a vision correction system. The fabrication of the Fresnel lenses and the experimental results of the visual correction systems show the superiority of the proposed 3D OPC method.

## 2. Computational Lithography Model with Subdomain Division

In the numerical model of 3D lithography, the intensity of the laser beam on the focal plane (surface of photoresist) is analytically expressed as an ideal Gaussian distribution; the thickness of the photoresist is assumed to be uniform; the reflection from the substrate is ignored in the thick film; and a series of optical effects, such as scattering and cavity formation, is also neglected. Thus, the lithographic process from CAD mask patterns to photoresist can be simplified into two numerical models: the optical scanning (aerial image $I_{a}$ formation) based on a programmable logic controller, and the resist effect (photoresist image $I_{p}$ formation) described by the Dill exposure model.

**Figure 2:**
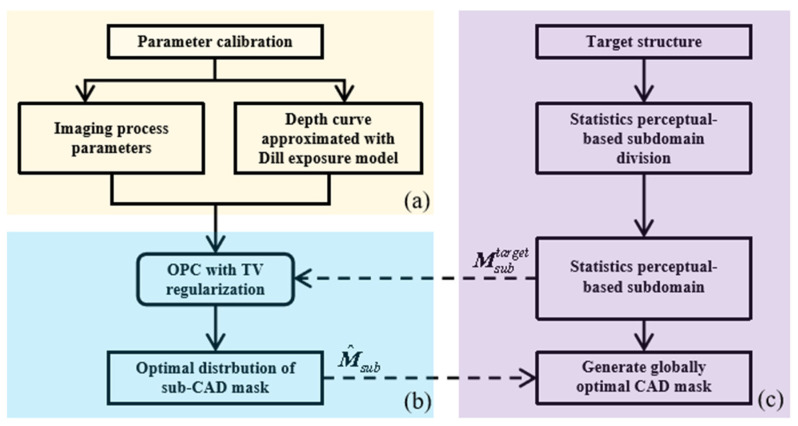
(**a**) Parameter calibration based on imaging model and Dill exposure approximation model. (**b**) The 3D OPC model with total variation (TV). (**c**) Subdomain segmentation and global recovery of mask.

As illustrated in Figure 2, the 3D OPC framework with a central symmetric centimeter target can be divided into three steps. Figure 2a depicts the parameter calibration of lithography, which mainly includes the light spot distribution and the static nonlinear response (depth curve) of the photoresist approximated by the Dill exposure model. In Figure 2b, a 3D OPC model with total variation (TV) is established, which penalizes discontinuities in all directions; through this TV term, the centrally symmetric exposure dose distribution is optimized. Subsequently, the target structure is segmented as $M_{sub}^{target}$ to ensure the efficiency of optimization, and the optimal distribution of the sub-CAD mask is synthesized through 3D OPC. The scheme of subdomain segmentation and the global recovery of the mask is shown in Figure 2c.

### 2.1. Forward Imaging Model

**Figure 3:**
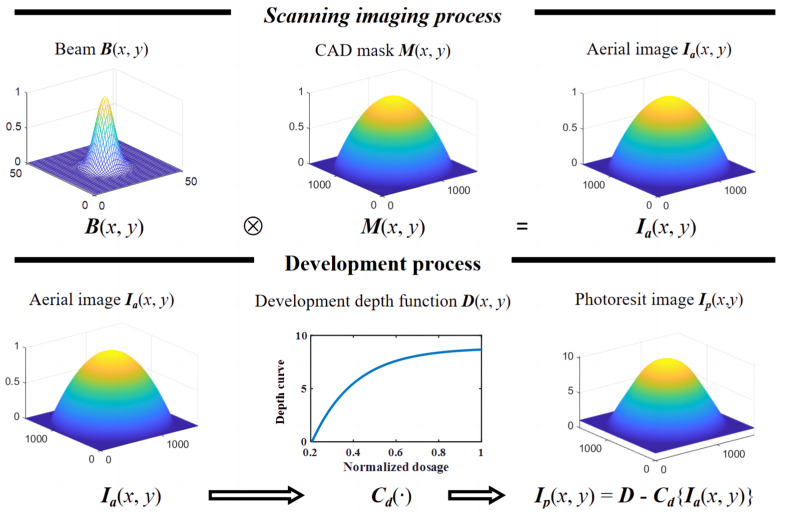
Numerical model of 3D lithography system, where the features on the CAD mask are transferred to the photoresist by a direct exposure and development process.

Under the assumption of a linear time-invariant system, the scanning imaging process of LDW is assumed to be incoherent imaging. As Figure 3 shows, given an input CAD mask *M*, the laser power is regulated according to the gray level of the mask, and the focused spot of different power is obtained through electrical modulation. After selective exposure by focusing spots of different power, an approximate aerial image of the photoresist surface can be calculated as

(1) $$I_{a}=M(x,y)⊗B(x,y)$$

in which ⊗ is the convolution operation and *B* is the Gaussian spot, which can be calculated by

(2) $$B\left(x,y\right)=\frac{2P}{\pi \omega_{0}^{2}}e^{-\frac{2(x^{2}+y^{2})}{\omega_{0}^{2}}}$$

where *P* and $\omega_{0}=\mathrm{FWHM}/\sqrt{2\ln2}$ are the total power and the waist of the beam.

**Figure 4:**
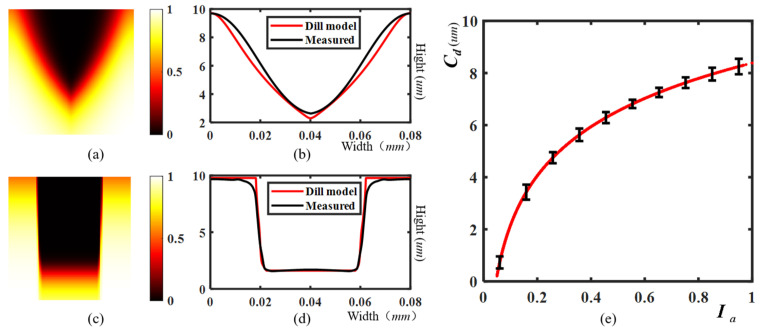
Comparison of Dill model and experiment, where (**a**,**c**) are the normalized PAC concentrations of resists with two different incident intensities $I_{a}$, and the corresponding resist curves computed by the Dill model and experiment are given in (**b**,**d**), respectively. (**e**) depicts the nonlinear relationship between normalized aerial image $I_{a}$ and photoresist depth $C_{d}\left(I_{a}\right)$.

As a semi-empirical model to describe the exposure process, the Dill exposure model leverages the photochemical reaction through the transport equation and nonlinear kinetic model. Therefore, the Dill model can accurately predict the formation of photoresist patterns and the generation of defects [22,23,24]. Such predictions can guide the optimization of the exposure parameters and process flow, hence improving the print fidelity. The numerical expression of the Dill exposure model can be described as

(3) $$\frac{\partial I(z,t)}{\partial z}=-I(z,t)[AM_{PAC}(z,t)+B]$$

(4) $$\frac{\partial M_{PAC}(z,t)}{\partial t}=-CI(z,t)M_{PAC}(z,t)$$

in which I(z,t) and $M_{PAC}(z,t)$ are the intensity and photoactive compound concentration (PAC) at the depth *z* in the resist. *A*, *B*, and *C* are the Dill parameters, which can be calculated by the transmittance of exposed and unexposed photoresists [22]. Although 3D lithography is discontinuous in the exposure time of the resist surface, the quasi-static approximation method based on the surface exposure is also useful [24]. Such prediction allows the optimization of the exposure parameters and process flow, thereby improving the print fidelity. To this end, it is possible to set the boundary condition of $I\left(0,\;0\right)=I_{a}$ and $M_{PAC}\left(0,\;0\right)=1$. Assuming that the development time is sufficient, and the properties of the photoresist are not damaged, an accurate development result can be obtained by setting the PAC concentration threshold $M_{tr}$. Figure 4 depicts the simulation results and experimental results of the Dill model. Figure 4a,c give the normalized PAC computed by different incident intensities $I_{a}$, with the resist curves computed by the Dill model and the experiment results given in Figure 4b,d. However, the iteration of partial differential equations based on Equations (3) and (4) is impractical in 3D OPC, because it brings huge computational resource requirements in derivation. Therefore, it is necessary to convert the 3D model into a 2D model.

Fortunately, Equations (3) and (4) were solved analytically by Herrick, and *B* can often be safely neglected while *A* >> *B* [46]. Thus, the relationship between the intensity and PAC can be approximated as

(5) $$I(z)=I(0)\cdot \frac{1-M(z)}{1-M(0)}=I(0)\cdot \frac{M(z)}{M(0)}e^{-Az}$$

Using the threshold method, assume that $I(z)=I_{tr}$ and $M(z)=M_{tr}$ are the development thresholds at depth *z*. The PAC at the top of photoresist $M(0)=M_{min}$ below the photochemical reaction threshold. Equation (5) is converted into Equation (6) by setting boundary condition $I\left(0\right)=I_{a}$:

(6) $$z=\frac{1}{A}\ln\left[\left(\frac{M_{tr}}{M_{\min}I_{tr}}\right)\cdot I_{a}\right]$$

Considering that the derivation of the photochemical reaction curve uses a series of approximate conditions, we have adopted a more general form in calibration:

(7) $$C_{d}\left(I_{a}\right)=z=a_{1}\cdot \ln\left(I_{a}+a_{2}\right)+a_{3}$$

The quasi-static approximation result can be calibrated as shown in Figure 4e. To this end, the photoresist image after development $C_{d}$ can be uniformly computed as

(8) $$I_{p}=T\left\{M\left(x,y\right)\right\}=D-C_{d}\left(I_{a}\right)$$

in which T{ ⋅ } represents the mapping relationship from the CAD mask and photoresist image, and ***D*** is the thickness of the photoresist.

### 2.2. Inverse Optimization Method with Statistics Subdomain Division

The goal of 3D OPC is to invert the optimal CAD mask $\tilde{M}$ and minimize the dissimilarity between desired target pattern $I_{0}$ and photoresist image $I_{p}$ over all pixels. The score function $S_{pe}$ employed in convex optimization can be computed by

(9) $$S_{pe}\left(M\right)=\frac{1}{2}{\left\|I_{p}-I_{0}\right\|}_{2}^{2}$$

in which ${\left\|\cdot \right\|}_{2}^{2}$ means taking the square of the l2 norm.

**Figure 5:**
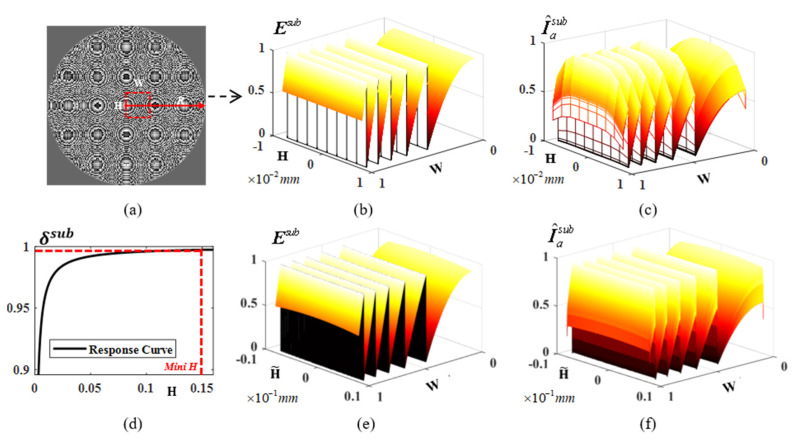
Subdomain division based on perceptual statistics. (**a**) Fresnel lens with radius *R* = 1 cm. (**b**) is the initial subdomain with the corresponding aerial image in (**c**). (**d**) gives the convolution response between the mask and aerial image. (**e**) shows the global performance of the convolution response. (**f**) is the optimal width and height of the subdomain.

However, the optimization is impracticable when the pixel number *N* reaches millions. An effective way to solve this problem is to divide the optimization into subdomains; then, the subdomains are optimized, and the global solution can be obtained by combing these subdomains solutions finally. Considering the uniqueness of the Fresnel design, the symmetrical structure is characterized through a limited area. Therefore, the inverted solutions in a limited subdomain $M^{sub}$ can perceive global solutions $\tilde{M}$. According to Equation (1), the optical proximity effect (OPE) always exists, and the distortion degree of the aerial image in the subdomain $I_{a}^{sub}$ is increased as subdomain $M^{sub}$ decreases. In Figure 5a, the subdomain mask with width W and height H is denoted as $M^{sub}$, and the mask intensity in polar coordinates is determined by the radius and independent of the angle. The 3D profile of the subdomain is given in Figure 5b, and the aerial image in the subdomain $I_{a}^{sub}$ has an obvious distortion at the edges when H<0.002mm. Figure 5c gives the normalized aerial image ${\hat{I}}_{a}^{sub}$ computed by

(10) $${\hat{I}}_{a}^{sub}(x,y)=M^{sub}(x,y)⊗B_{sum}^{-1}\left(x,y\right)$$

where $B_{sum}^{-1}\left(x,y\right)=B\left(x,y\right)/\sum_{\left(x,y\right)}B\left(x,y\right)$.

According to the linear characteristic of the convolution operation, ${\hat{I}}_{a}^{sub}(x,y)\approx M^{sub}(x,y)$ in the fully convoluted region, and ${\hat{I}}_{a}^{sub}(x,y)\ll M^{sub}(x,y)$ in the incompletely convoluted region. Thus, the responses of convolution on subdomain $M^{sub}$ can be computed to describe the overall response relationship. To quantify the extent of distortion, we utilize the linear characteristic of the convolution operation. The response relationship between ${\hat{I}}_{a}^{sub}$ and $M^{sub}$ is defined as

(11) $$\delta^{sub}=\frac{1}{kh\cdot kw}\sum_{x=1}^{kh}\sum_{y=1}^{kw}{\hat{I}}_{a}^{sub}(x,y)\cdot {\left[M^{sub}(x,y)+\varepsilon_{\delta }\right]}^{-1}$$

in which *kh* and *kw* represent the pixel number in the *x*/*y* direction of subdomain $M^{sub}$, and Figure 5d shows the relationship between response $\delta^{sub}$ and height **H**. Thus, the optimal height $\tilde{H}$ = 0.16 mm is determined by $\delta^{sub}=0.997$ according to the 3δ statistic principle. The subdomain and corresponding aerial image of subdomain ${\hat{I}}_{a}^{sub}$ are shown in Figure 5e,f. Similarly, the optimal width $\tilde{W}=R+\tilde{H}$ can be obtained from the circular symmetry property. The region to be optimized and the corresponding discrete pixel matrix are compressed to $\tilde{W}\cdot \tilde{H}\cdot {\left(2\cdot R\right)}^{-2}$. Hence, when optimizing a Fresnel lens with a diameter of 10 mm, we can effectively reduce the memory requirement to less than 1% by selecting $\tilde{H}$ = 0.16 mm and **R** = 5 mm as the appropriate parameters. This optimization allows us to strike a favorable balance between computational efficiency and maintaining the desired level of accuracy in the lens design process.

Without loss of generality, the optimization scheme is carried out in the Cartesian coordinate system, and the discontinuity of the optimal ${\hat{M}}^{sub}$ should be penalized by total variation (TV) in each radial direction. It is worth noting that we use the square of TV to avoid arithmetic errors. Therefore, the optimization problem can be formulated as

(12) $$\begin{array}{cc} \mathrm{minimize} & \;\;S=\lambda_{1}S_{pe}\left(M^{sub}\right)+\lambda_{2}S_{TV}^{2}\left(M^{sub}\right)\\ \mathrm{subject}\;\mathrm{to} & \;\;M^{sub}=\left(1+\cos\omega^{sub}\right)/2 \end{array}$$

where $\lambda_{1}=S_{pe}/\left|S_{pe}\right|$ and $\lambda_{2}=S_{TV}^{2}/\left|S_{TV}^{2}\right|$ represent the weights of $S_{pe}$ and $S_{TV}^{2}$, and $S_{TV}^{2}\left(M^{sub}\right)$ is given by

(13) $$S_{TV}^{2}\left(M^{sub}\right)={\left\|\nabla_{x}M^{sub}(x,y)\right\|}_{2}^{2}+{\left\|\nabla_{y}M^{sub}(x,y)\right\|}_{2}^{2}$$

in which $\nabla_{x}M^{sub}(x,y)$ and $\nabla_{y}M^{sub}(x,y)$ denote the gradients of $M^{sub}(x,y)$ in the *x* and *y* directions:

(14) $$\{\begin{array}{c} \nabla_{x}M^{sub}(x,y)=\frac{M^{sub}(x+1,y)-M^{sub}(x-1,y)}{2}\\ \nabla_{y}M^{sub}(x,y)=\frac{M^{sub}(x,y+1)-M^{sub}(x,y-1)}{2} \end{array}$$

Therefore, the optimal subdomain ${\hat{M}}^{sub}$ with respect to ω can be solved numerically by the steepest gradient descent (SGD) method, with the update rules given by

(15) $$\omega_{t+1}^{sub}=\omega_{t}^{sub}-\eta \cdot \partial S_{t}/\partial \omega_{t}^{sub}$$

where η is set to a reasonable value and ω will converge under the impetus of ∂S/∂ω. The calculation details of ∂S/∂ω can be found in [47]. It is worth noting that Equation (14) is converted into matrix multiplication to realize the solution of the partial differential.

### 2.3. Accelerated Algorithms

Although Equation (13) gives a stable and reliable optimization scheme, the convergence speed suffers in the case of multi-task optimization (optimization with constraints), which brings inconsistency in time steps η, as we have demonstrated [25,26]. In particular, 3D OPC is an ill-posed non-convex optimization problem. In addition to considering the optimization rate, it is crucial to prevent the optimization from converging to local minima. Thus, more robust Adam optimizer is employed in this paper [48], where the finite difference scheme at time-step *t* + 1 is

(16) $$\omega_{t+1}^{sub}=\omega_{t+1}^{sub}-\eta \cdot {\hat{m}}_{t}/\left(\sqrt{{\hat{v}}_{t}}+\varepsilon \right)$$

in which η=0.01 is the global learning rate. ${\hat{m}}_{t}=m_{t}/\left(1-\beta_{1}^{t}\right)$ is the first bias-corrected moment estimate of the first bias moment $m_{t}=\beta_{1}\cdot m_{t-1}+\left(1-\beta_{1}\right)\cdot g_{t}$. $\nu_{t}=\beta_{2}\cdot \nu_{t-1}+\left(1-\beta_{2}\right)\cdot g_{t}^{2}$ is the second bias-corrected moment estimate of the second bias moment $\nu_{t}=\beta_{2}\cdot \nu_{t-1}+\left(1-\beta_{2}\right)\cdot g_{t}^{2}$. The gradient is set as $g_{t}=\partial S/\partial \omega$, with the global decay rates $\beta_{1}=0.99$ and $\beta_{2}=0.999$. The additional term $\varepsilon =10^{-8}$ is included to ensure that the denominator is not zero.

On the other hand, the Fourier transform operation is applied to accelerate the convolution operation:

(17) $$M^{sub}(x,y)⊗B\left(x,y\right)=F^{-1}\left\{F\left\{M^{sub}(x,y)\right\}⊙F\left\{B\left(x,y\right)\right\}\right\}$$

in which F{ ⋅ } and $F^{-1}\left\{\;\cdot \;\right\}$ represent the forward and inverse Fourier transform operations, respectively, and ⊙ denotes the entry-by-entry multiplication operation.

## 3. Fabrication of the Fresnel Lens

### 3.1. Design of the Fresnel Lens

Similar to our previous work, a hyperbolic lens is designed in advance [15,16]:

(18) $$\frac{{\left(z-a\right)}^{2}}{a^{2}}-\frac{r^{2}}{b^{2}}=1$$

in which *a* = −7.475 mm and *b* = 8.356 mm are the semi-perimeters of the real and imaginary axes of the hyperbola, respectively. The conical coefficient and radius of curvature being k=−2.5 and R=9.34 mm, the focal length and f-number of the lens are *f* = 23.35 mm and *f*/# = 23.35 mm. Thus, the depth of the Fresnel lens grooves, with a constant height, can be simplified as [35,36]

(19) $$z_{i+1}=R-\sqrt{R^{2}-{\left(ip\right)}^{2}}$$

where *i* and *p* are the point coordinate and pitch size of the groove.

### 3.2. Equipment and Process Parameters

As illustrated in Figure 1a, the lithography device based on the 4096-order electrical modulation is the PicoMaster-100 (Raith/4PICO Litho, Dortmund, Germany). The full width at half maximum (FWHM) of the Gaussian beam profile is 850 nm, the scan speed and step size are v = 100 mm/s and s = 200 nm, and the minimum and maximum exposure doses are 50 mj/cm^2^ and 1000 mj/cm^2^, respectively. The customized 10-inch glass substrate is covered by the AZ4562 photoresist (PuZhao Display Equipment Co., Ltd., Changsha, China) with a thickness of 10 μm. The development process uses 25% KOH solvent for 120 s at a room temperature of 22 °C.

### 3.3. Simulation Parameters of 3D OPC

The parameters used for the numerical simulations are as follows: full width at half maxima FWHM = 850 nm; spatial resolution Δx = Δy = 200 nm/pixel; Dill parameters A = 0.45, B = 0.022, C = 0.017, and $M_{tr}=0.6$; quasi-static approximation coefficient a1 = 3.38, a2 = 47.76, and a3 = −17.6; height of subdomain $\tilde{H}=0.16$ mm. All computations are performed on an RTX 3090 GPU (NVIDIA, Santa Clara, CA, USA) with 24 GB memory.

### 3.4. Results and Discussion

**Figure 6:**
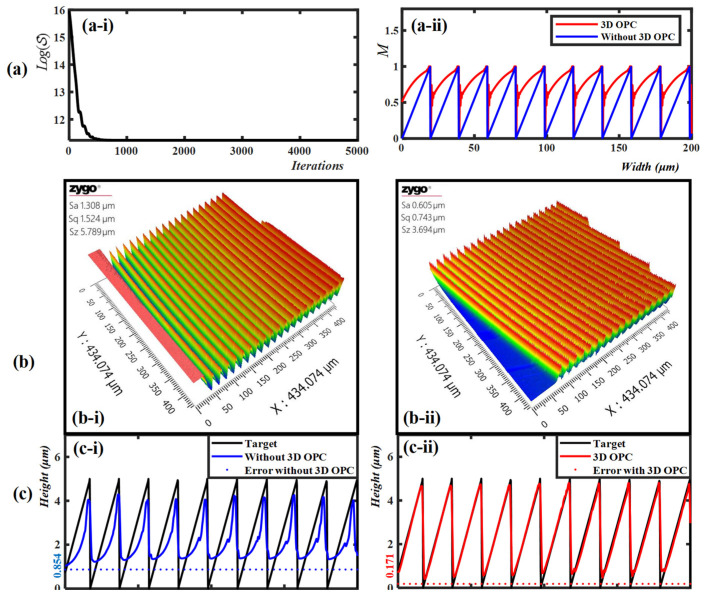
Simulation and experimental results with last 10 grooves. Rows (**a**–**c**): the simulation results and the lithography results before and after 3D OPC, respectively. (**a-i**) Convergence performance of score function. (**a-ii**) The cross-section profile of CAD mask before (blue line) and after (red line) 3D OPC. (**b-i**,**b-ii**) shows the lithography result before and after 3D OPC, with the 3D profile measured by an optical profiler. (**c-i**) Schematic illustration of target profile (black line) and the profiles before (blue line) 3D OPC, where the MSE error between the target profile and lithographic profiles (blue horizontal dotted line) is 0.854 μm. (**c-ii**) Schematic illustration of target profile (black line) and the profiles after (red line) 3D OPC, where the MSE error between the target profile and lithographic profiles (read horizontal dotted line) is 0.171 μm.

The optimization and experimental results are given in Figure 6. Figure 6a–c depict the simulation results and the lithography results before and after 3D OPC. Figure 6(a-i) gives the convergence performance of the 3D OPC method; the score function S is reduced from 8.5 million to 76 thousand. Figure 6(a-ii) shows a cross-section of the CAD mask of the last 10 grooves, where the blue and red lines represent the CAD mask before and after 3D OPC. The CAD mask values based on 3D OPC are increased due to the resist effect, and the discontinuities of the edges are searched to deal with the optical proximity effect (OPE). Figure 6b gives the lithography result before (Figure 6(b-i)) and after (Figure 6(b-ii)) 3D OPC, which was measured by an optical profiler (ZYGO Co., Ltd., Nexview NX2, Middlefield, CT, USA). Figure 6(c-i) illustrates the target structure (black line) and the lithographic structures before (blue line) 3D OPC, where the MSE error (blue horizontal dotted line) between the target one (black line) and the lithographic one (read line) is 0.854 μm. Figure 6(c-ii) illustrates the target structure (black line) and the lithographic structures after (red line) 3D OPC, where the MSE error (red horizontal dotted line) between the target one (black line) and the lithographic one (read line) is 0.171 μm. According to the experiments, the MSE error of the optimized profile is reduced to 79.98% compared with the profile without 3D OPC, which confirms the superiority of the proposed method. It is worth noting that the proposed memory compression method is based on the properties of structural profiles and reverse optimization algorithms, so it is suitable for different lithography processes and lithography models.

**Figure 7:**
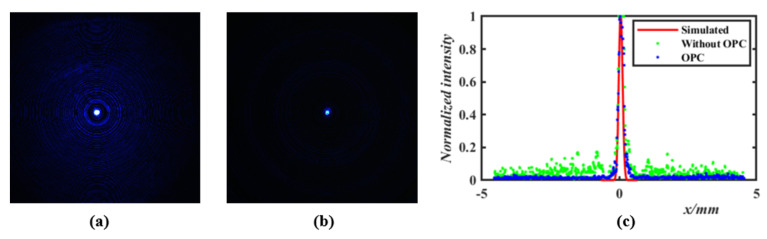
Schematic illustration of focusing spot, with the wavelength 450 nm. (**a**) Focal spot of Fresnel lens without 3D OPC. (**b**) Focusing spot of Fresnel lens based on 3D OPC. (**c**) Intensity profile of focal spot image and simulated intensity profile.

In the process of spot measurement, it is necessary to move the Fresnel lens continuously in the *z* direction to determine the focal plane at the best focusing quality, so as to obtain the spot at the focal plane. Figure 7 shows a comparison of the focusing spot of Fresnel lenses (before and after 3D OPC). When the focal length is *f* = 32.55 mm before 3D OPC, there are multiple undesired peaks with considerable intensity in the image. The focal spot image of the Fresnel lens without 3D OPC is given in Figure 7a; the corresponding f-number is *f*/# = 32.55. Figure 7b depicts the focus spot of the Fresnel lens based on 3D OPC, with a clear peak optimized by 3D OPC, and the improved performance is evidenced by the focus and f-number of *f* = 24.25 mm and *f*/# = 2.425. The comparison of spot profiles given in Figure 7c, where the simulated profile and the profiles before and after 3D OPC optimization are represented by red solid lines, green dashed lines, and blue dashed lines. The actual focal length of the optimized Fresnel lens is close to the design focal length of *f* = 23.35 mm, and the actual focal length of the optimized Fresnel lens is much longer than the designed focal length because of the concave distortion.

## 4. Application of the Fresnel Lens

### 4.1. Fabrication of the Transferred Fresnel Lens

We convert convex Fresnel lenses into concave Fresnel lenses for myopia correction. The transfer process from photoresist to PDMS is divided into three steps. The first step is to mix PDMS (Sylgard 184, Dow Corning, Midland County, MI, USA) with a curing agent at a weight ratio of 1:10 and to remove bubbles through a vacuum environment. The second step is to apply PDMS on the Fresnel lens and use the spin coating method to prepare a mixture film. The spin coating process includes two stages: the film is spun with the speed of 400 rpm for 20 s in the first stage; then, it is spun with the speed of 700 rpm for 40 s in the second stage. In the third step, the PDMS mixture coating on the zoom MLA is fed into an oven at 60 for 7 h. After the baking process, the PDMS mixture is peeled off from the Fresnel lens.

### 4.2. Vision-Correcting System

In the experiments with the vision-correcting system, a 4× objective lens (Daheng, GCO-2121, Beijing, China) is used to represent the human crystalline lens; a CCD (Daheng, MER-2000-19U3C-L, acquisition frame rate 25 Hz, exposure time 50,000 μs) is used to observe the image, which is simply approximated as an image on the retina. A white light source is selected to imitate natural light imaging and used for testing on the 1951 USAF resolution test card. The color image target is displayed on the OLED screen. The adjustment of the object (mask) and the image distances is realized by two three-dimensional motion platforms (Daheng, GCM-901602 M).

### 4.3. Results and Discussion

**Figure 8:**
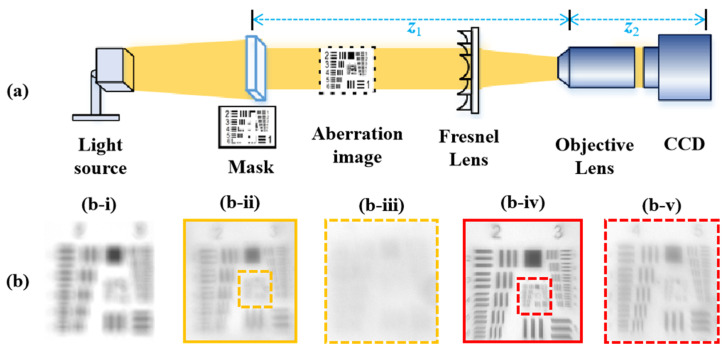
(**a**) Schematic illustration of an experiment for vision correction. (**b**) Comparisons of best imaging performance with 1951 USAF resolution test chart. (**b-i**) is optical image without lens, (**b-ii**) is the optical image corrected by transferred lens (without 3D OPC), (**b-iii**) is the zoomed-in image of group 5 element of (**b-ii**), (**b-iv**) is the optical image corrected by transferred lens (3D OPC), (**b-v**) is the zoomed-in image of group 5 element of (**b-iv**).

In this paper, we consider the correction of myopia, where the retina receives blurred images in the distance. Therefore, a concave lens needs to be placed in front of the human eye to extend the focal length. With the transfer process provided in Section 3.4, the vision correction lens (transferred Fresnel lens) is used to extend the focal length. Figure 8a shows the optical system for vision correction. The distance between the mask and objective lens is defined as z1, and the distance between the objective lens and CCD is defined as z2. In practice, the vision correction approximation experiment can be divided into three steps: normal vision imaging, myopia vision imaging, and vision correction imaging.

The first step is to obtain normal vision imaging without using a vision correction lens: the positions of the mask and objective lens are continuously adjusted to enable the CCD to capture a clear image. During this process, white light illumination is used, and a 1951 USAF resolution test card is used as the mask, resulting in z1 = 40 mm, z2 = 90 mm. The second step is to obtain myopia vision imaging without using a vision correction lens: we stabilize the position of the objective lens and CCD and move the mask away from the objective lens to allow the CCD to receive aberration images. Figure 8(b-i) gives the aberration images received by CCD, where the distance between the mask and objective lens is z1 = 80 mm. The third step is to obtain vision correction imaging by using a vision correction lens: we place a transferred Fresnel lens in front of the objective lens to extend the focal length of the optical path. We continuously adjust the position of the vision correction lens to achieve the best image quality received by the CCD. Based on a visual correction lens without 3D OPC, the corrected image, as shown in Figure 8(b-ii), is blurry, and the zoomed-in image of group 5 is given in Figure 8(b-iii). It can be observed that only the features in element 2 of group 3 can be distinguished. Figure 8(b-iv) shows the imaging correction result of the vision correction lens optimized by 3D OPC, with the zoomed-in image of group 5 elements shown in Figure 8(b-v), and the equivalent corrected resolutions are 14.25 lp/mm (group 3, element 6) and 32 lp/mm (group 5, element 6). Although the blurred grayscale images of group 5 cannot represent the absolute resolution of corrected vision, all of the resolution features are captured.

**Figure 9:**
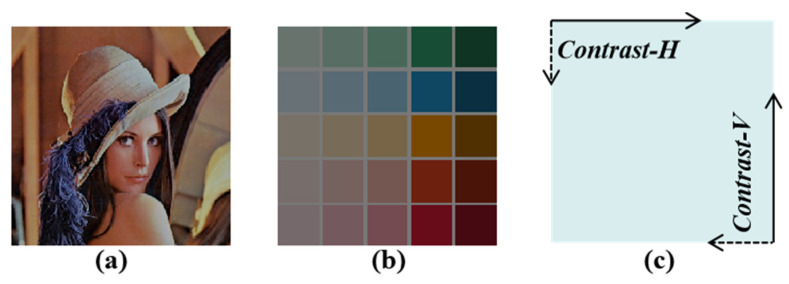
(**a**) The portrait target. (**b**) The color blocks target. (**c**) Schematic diagram of contrast calculation.

Through the above operations, the optimal placement of vision correction lenses can be ensured. Thus, colorful image masks can be employed in the imaging system to quantify the imaging performance. The portrait and blocks shown in Figure 9a,b are displayed on the OLED screen. The contrast is calculated as shown in Figure 9c, i.e., the contrast-H and -V of the whole image are averaged horizontally and vertically.

**Figure 10:**
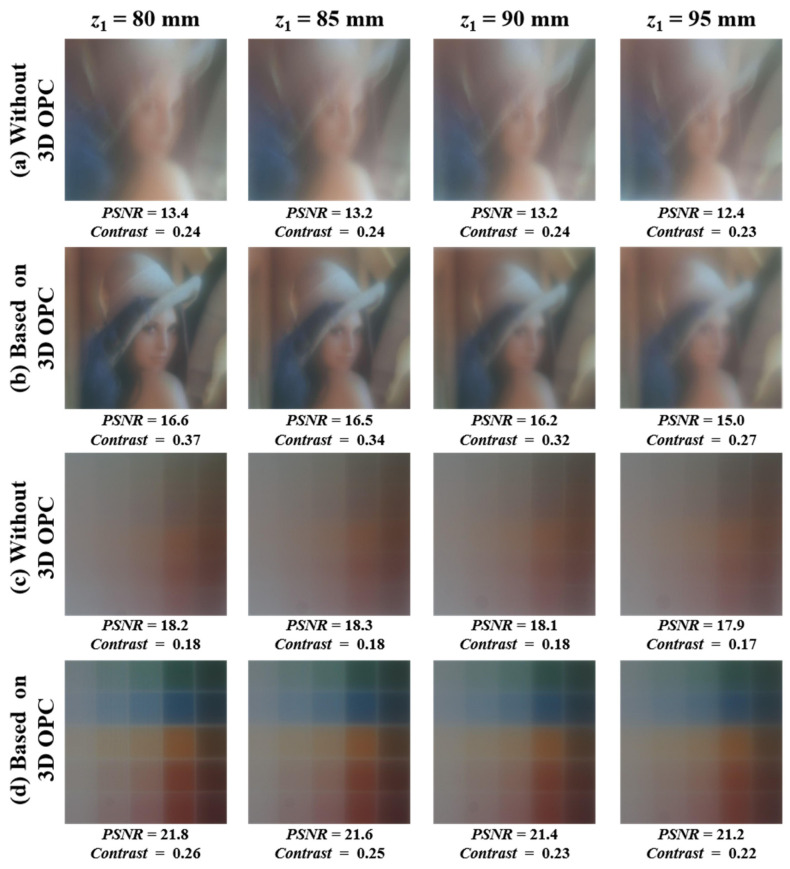
Experimental results of imaging correction for vision correction lenses. Columns from left to right: the imaging results at z1 = 80, 85, 90, and 95 mm, respectively. Rows (**a**,**b**) give the imaging correction results using portrait mask. Rows (**c**,**d**) show the imaging correction results using color block mask.

Figure 10 depicts the imaging correction results based on vision correction lenses. The columns from left to right show the imaging results at z1 = 80, 85, 90, and 95 mm. With the portrait mask employed in rows (a) and (b), the imaging results based on the vision correction lens (before and after 3D OPC) are compared. In row (a) of Figure 10, the imaging results are corrected by the Fresnel lens without 3D OPC, where PSNR = 13.4, 13.2, 13.2, 12.4 dB and contrast = 0.24, 0.24, 0.24, 0.23 at z1 = 80, 85, 90 and 95 mm, respectively. Row (b) gives the imaging correction results for the Fresnel lens based on 3D OPC, with the improved PSNR = 16.6, 16.5, 16.2, 15.0 dB and contrast = 0.37, 0.34, 0.32, 0.27. Similarly, the imaging correction results with the block target are depicted in rows (c) and (d). The PSNR = 18.2, 18.3, 18.1, 17.9 dB and contrast = 0.18, 0.18, 0.18, 0.17 based on a Fresnel lens without 3D OPC are given in row (c). The improved performance of corrected imaging is shown in row (d), with the PSNR = 21.8, 21.6, 21.4, 21.2 dB and contrast = 0.26, 0.25, 0.23, 0.22. Although the PSNR and contrast calculated based on different complexity masks are different, the Fresnel lens based on 3D OPC greatly improves the imaging performance of the vision correction system. The average PSNR and the average contrast (computed based on different positions z1) before and after 3D OPC are presented in Table 1. Conservatively quantified, we only take smaller values. Compared with the imaging results formed by the vision correction lens without 3D OPC, the average PSNR and average contrast with the 3D OPC-based vision correction lens are improved by 18.92% and 36%, respectively. Without loss of generality, we take the minimum improvement ratio for quantification.

## 5. Conclusions

In this paper, an improved 3D OPC method based on statistical principles is proposed, which reduces the memory requirement to less than 1% and realizes 3D OPC in the micron to millimeter scale. With the quasi-static approximated Dill exposure model embedded in 3D OPC, the 3D model is approximated as a 2D model and transformed into a differentiable scheme. Benefitting from the proposed methods, the CAD mask of the Fresnel lens is inverted within 10 min, and the experimental results show that the reduction in the geometric profile error after optimization is 79.98%. With a vision-correcting lens transferred from a 3D OPC-based Fresnel lens by PDMS, the resolution of the vision-correcting system reaches 32 lp/mm. Compared with the transferred vision correction lens without 3D OPC, the PSNR and average image contrast of the vision correction system are improved by 18.92% and 36%, respectively. It should be noted that the image quality of the optimized Fresnel lens is still insufficient. This is due to various factors contributing to the generation of aberrations, as well as inherent limitations in the device’s imaging characteristics, particularly evident in Fresnel lenses. This paper compares the image quality of the Fresnel lens before and after optimization in terms of preparation and validates the reliability of the proposed algorithm. Future work will involve employing a 3D OPC algorithm to optimize the achromatic lens, with the goal of enhancing the PSNR and contrast of the imaging. These improvements are envisioned to have potential applications in vision correction.

## Figures and Tables

**Table 1:** Performance of mask imaging based on vision correction lens before and after 3D OPC.

Mask	Vision Correction Lens without 3D OPC		Vision Correction Lens with 3D OPC		Improvement	
	PSNR (dB)	Contrast	PSNR (dB)	Contrast	PSNR	Contrast
Portrait	13.0	0.24	16.1	0.32	23.3%	37.29%
Color Blocks	18.1	0.18	21.5	0.24	18.92%	36%

## Data Availability

The data presented in this study are available from the corresponding author on request. The data are not publicly available due to privacy.
